# Association Between Graphic Health Warning Labels on Cigarette Packs and Smoking Cessation Attempts in Korean Adolescent Smokers: A Cross-Sectional Study

**DOI:** 10.3389/fpubh.2022.789707

**Published:** 2022-02-11

**Authors:** Hye Jin Joo, Jae Hong Joo, Seung Hoon Kim, Eun-Cheol Park, Sung-In Jang

**Affiliations:** ^1^Department of Public Health, Graduate School, Yonsei University, Seoul, South Korea; ^2^Institute of Health Services Research, Yonsei University, Seoul, South Korea; ^3^Department of Preventive Medicine, Yonsei University College of Medicine, Seoul, South Korea

**Keywords:** smoking, graphic health warning labels, smoking cessation, adolescent smokers, Korean adolescents, Korea Youth Risk Behavior Web-based Survey

## Abstract

Graphic health warning labels on cigarette packs inform smokers about the health risks associated with tobacco smoking. Adolescents are generally the main targets to influence by graphic health warning labels. This study investigated the association between graphic health warning labels on cigarette packs and attempts to quit smoking in South Korean adolescents. This cross-sectional study used data from the 2017 to 2019 Korea Youth Risk Behavior Web-based Survey, using multiple logistic regression for the analysis. The study population comprised 11,142 adolescents aged 12–18 years. The outcome variable was attempts to quit smoking among adolescent smokers who had seen graphic health warning labels. Attempts to quit smoking were higher among adolescent smokers who had seen graphic health warning labels compared to those who had not {boys, odds ratio (OR) = 1.72 [95% confidence interval (CI), 1.48–2.00]; girls, OR = 1.74 (95% CI, 1.33–2.28)}. The correlation was greater for adolescents who thought about the harm of smoking [boys, OR = 1.86 (95% CI, 1.60–2.16); girls, OR = 1.85 (95% CI, 1.41–2.43)] and the willingness to quit [boys, OR = 2.03 (95% CI, 1.74–2.36); girls, OR = 2.04 (95% CI, 1.55–2.68)] after seeing graphic health warning labels. Our findings indicate that graphic health warning labels on cigarette packs have the potential to lower smoking intentions of adolescents. We suggest that the use of graphic health warning labels is an effective policy-related intervention to reduce smoking in South Korean adolescents.

## Introduction

Smoking is a critical public health concern worldwide and a risk factor for various non-communicable diseases (1) such as cancer, cardiovascular and respiratory diseases (2) and mental health (3, 4). Globally, more than 8 million smoking-related deaths are reported annually; and more than 7 million of those are attributed to direct tobacco use (5). The smoking prevalence in Korean adolescents was 21.9% among boys and 7.1% among girls in 2016. The average age of smoking initiation among Korean adolescents was 12.7 years, which was lower than the international average age of 13–15 years (6). Additionally, the proportion of adult smokers who started smoking under the age of 18 was 45.6% for men and 33.4% for women in 2012 (7). The earlier the smoking initiation, the greater the nicotine addiction (8). Smoking during adolescence is closely associated with negative effects, including impaired physical and mental development and unhealthy behaviors. Therefore, the decrease in the age of onset of adolescent smoking emphasizes the need to regulate access to tobacco products.

Many countries have implemented various tobacco control policies to prevent smoking among children and adolescents. Tobacco control policies for adolescents conventionally aim at reducing the initiation and frequency of smoking and encouraging quitting (9). For example, policies such as increasing the price of tobacco products, smoking prevention education, smoke-free legislation, and mass media campaigns can help prevent initiation of smoking and reduce the prevalence of smoking among adolescents (10, 11).

The graphic health warning labels (GHWL) on cigarette packs are the most common means of alerting smokers about the health risks associated with smoking. These vivid pictorial representations of risks associated with smoking are more effective in raising health risk awareness and motivating smokers to quit than providing text-based labels (12–14). The World Health Organization's (WHO) Framework Convention on Tobacco Control (FCTC) has invited member countries to implement measures to reduce the demand for tobacco products (15). Accordingly, the implementation of GHWL on cigarette packs, in most countries, has shown a significant effect in reducing the smoking rate and preventing smoking initiation among adolescents. Previous studies have shown that pictorial warnings on cigarette packs can significantly increase people's awareness regarding harm from tobacco use (2). Although South Korea implemented GHWL on cigarette packaging as a policy in December 2016 (16), and evaluations of warning exposure and the effects of GHWL have been adequately conducted, investigations on GHWL policies targeting adolescents are sparse. Since one of the purposes of implementing GHWL is to prevent smoking among adolescents, it is important to assess whether GHWL affect adolescents' perceptions and attitudes toward smoking.

In this study, we aimed to comprehensively examine how GHWL influence attempts to quit smoking, to develop effective health policies for successful smoking cessation in Korean adolescents. We hypothesized that GHWL would be highly associated with attempts to quit smoking among adolescents.

## Materials and Methods

### Data Source and Study Population

We used data from the Korea Youth Risk Behavior Web-based Survey (KYRBWS) (17) 2017–2019. The KYRBWS was designed as a cross-sectional study and comprised a nationwide web-based survey of Korean adolescents' health behaviors. This survey has been conducted annually by the Korea Centers for Disease Control and Prevention (KCDC) to evaluate health indicators for promoting youth health since 2005. Participants were students in grades 7–12 in South Korea. The study design included multistage sampling, stratification, and clustering. The samples were stratified by 44 regional and school type variables. The validity and reliability of the data collected have been confirmed by previous studies (18, 19). KYRBWS data provide anonymous, secondary data that are publicly available for scientific use. The initial study population comprised 1,79,619 adolescents (2017, *n* = 62,276; 2018, *n* = 60,040; 2019, *n* = 57,303). Among them, we used a sample of adolescents who reported smoking a cigarette—including conventional cigarettes, electronic cigarettes, and heated tobacco products—at least once (*n* = 25,752), which was then stratified by sex (boys, *n* = 18,789; girls, *n* = 6,963). We excluded participants who did not respond to the questions or those whose data contained missing values for variables. Finally, 11,142 adolescents (boys, *n* = 8,147; girls, *n* = 2,995) were included as a representative sample in the study.

### Attempt to Quit Smoking

The main objective of this study was to analyze attempts to quit smoking. The survey question was as follows: “During the past 12 months, have you ever attempted to quit smoking?” Attempts to quit smoking were divided into two groups according to “yes” or “no” responses to the questions.

### Graphic Health Warning Labels

The main exposure of interest was recognition of GHWL. The survey question was as follows: “During the past 30 days, did you see a graphic health warning on tobacco products?” The responses were classified as “yes” or “no”. Participants who responded positively to seeing GHWL in the past 30 days were asked two questions: “When you saw the graphic health warning, did you think that you should not smoke?” and “When you saw the graphic health warning, did you think that smoking is harmful to health?” There were four responses for each question: “never” “sometimes” “often” and “always”. Based on survey responses, we reclassified the participants into two groups as follows: yes (sometimes, often, or always) and no (never).

### Covariates

Demographic, socioeconomic, and health-related factors were included as covariates in the study. The demographic and socioeconomic covariates included age (12–18), region (metropolitan, urban, rural), grade (middle school, high school), living with family (yes, no), household income level (low, medium, high), school record (good, average, poor). Household income level and grade was determined by the self-reported questions about household income level and GPA score. The health-related covariates included smoking amount per day ( ≤ 1, 2–9, ≥10), smoking onset (elementary school, middle school, high school), passive smoking at home (yes, no), alcohol drinking frequency (frequently, occasionally, never), physical activity exceeding 60 min per day (5–7 days a week, 1–4 days a week, none), stress (high, low, none), and depression (yes, no).

### Statistical Analyses

All analyses were stratified by sex, as women may be reluctant to disclose their smoking habits (20). Descriptive analysis and chi-square tests were performed to examine the distribution of the general characteristics of the study population. The statistical significance level was defined as *p*-value <0.05. Multiple logistic regression analysis was performed to determine odds ratios (ORs) and 95% confidence intervals (CIs) to identify the association between GHWL and attempts to quit smoking after adjusting for all covariates. Subgroup analysis was conducted to investigate this association according to stratified smoking amount per day, age at smoking onset, passive smoking at home, drinking frequency, and physical activity using a multiple logistic regression analysis. All analyses were performed using the SAS 9.4 software (SAS Institute, Cary, NC, USA).

## Results

The general characteristics of the sex-stratified study participants are presented in Table 1. Of 11,142 participants, 8,147 were boys and 2,995 were girls. Overall, 7,038 (86.4%) of the boys and 2,679 (89.4%) of the girls reported having seen GHWL on cigarette packs. Among them, 5,091 (72.3%) of the 7,038 boys and 1,901 (71.0%) of the 2,179 girls had attempted to quit smoking.

**Table 1:** General characteristics of the study population.

**Variables**	**Attempts to quit smoking**													
	**Boys (*****n*** **=** **8,147)**							**Girls (*****n*** **=** **2,995)**						
	**Total**		**Yes**		**No**		*P-*value^*^	**Total**		**Yes**		**No**		*P-*valu^e*^
	***N***	**%**	***N***	**%**	***N***	**%**		***N***	**%**	***N***	**%**	***N***	**%**	
Graphic health warning labels							<0.0001							<0.0001
Have not seen	1,109	13.6	657	59.2	452	40.8		316	10.6	188	59.5	128	40.5	
Have seen	7,038	86.4	5,091	72.3	1,947	27.7		2,679	89.4	1,901	71.0	778	29.0	
Smoking amount per day							<0.0001							<0.0001
≤ 1	2,199	27.0	1,424	64.8	775	35.2		1,035	34.6	657	63.5	378	36.5	
2~9	4,148	50.9	3,140	75.7	1,008	24.3		1,496	49.9	1,127	75.3	369	24.7	
≥10	1,800	22.1	1,184	65.8	616	34.2		464	15.5	305	65.7	159	34.3	
Smoking onset							<0.0001							0.0006
Elementary school	2,014	24.7	1,371	68.1	643	31.9		509	17.0	339	66.6	170	33.4	
Middle school	4,997	61.3	3,630	72.6	1,367	27.4		1,814	60.6	1,312	72.3	502	27.7	
High school	1,136	13.9	747	65.8	389	34.2		672	22.4	438	65.2	234	34.8	
Secondhand smoking at home							0.1213							0.4946
Yes	3,229	39.6	2,247	69.6	982	30.4		1,446	48.3	1,000	69.2	446	30.8	
No	4,918	60.4	3,501	71.2	1,417	28.8		1,549	51.7	1,089	70.3	460	29.7	
Age							<0.0001							0.0240
12	84	1.0	56	66.7	28	33.3		43	1.4	24	55.8	19	44.2	
13	366	4.5	264	72.1	102	27.9		187	6.2	136	72.7	51	27.3	
14	782	9.6	582	74.4	200	25.6		377	12.6	273	72.4	104	27.6	
15	1,275	15.6	938	73.6	337	26.4		534	17.8	384	71.9	150	28.1	
16	1,946	23.9	1,415	72.7	531	27.3		659	22.0	471	71.5	188	28.5	
17	2,364	29.0	1,603		761			825	27.5	564	68.4	261	31.6	
18	1,330	16.3	890	66.9	440	33.1		370	12.4	237	64.1	133	35.9	
Region							0.2074							0.7709
Metropolitan	3,440	42.2	2,391	69.5	1,049	30.5		1,230	41.1	849	69.0	381	31.0	
Urban	3,944	48.4	2,813	71.3	1,131	28.7		1,513	50.5	1,063	70.3	450	29.7	
Rural	763	9.4	544	71.3	219	28.7		252	8.4	177	70.2	75	29.8	
Grade							0.0003							0.2324
Middle school	1,760	21.6	1,303	74.0	457	26.0		848	28.3	605	71.3	243	28.7	
High school	6,387	78.4	4,445	69.6	1,942	30.4		2,147	71.7	1,484	69.1	663	30.9	
Living with family							0.0026							0.1683
Yes	7,478	91.8	5,310	71.0	2,168	29.0		2,730	91.2	1,914	70.1	816	29.9	
No	669	8.2	438	65.5	231	34.5		265	8.8	175	66.0	90	34.0	
Household income							0.8755							0.8730
Low	1,532	18.8	1,086	70.9	446	29.1		758	25.3	531	70.1	227	29.9	
Medium	3,399	41.7	2,403	70.7	996	29.3		1,305	43.6	914	70.0	391	30.0	
High	3,216	39.5	2,259	70.2	957	29.8		932	31.1	644	69.1	288	30.9	
School record							0.0007							<0.0001
Well	2,052	25.2	1,380	67.3	672	32.7		658	22.0	416	63.2	242	36.8	
Common	1,997	24.5	1,427	71.5	570	28.5		605	20.2	420	69.4	185	30.6	
Poor	4,098	50.3	2,941	71.8	1,157	28.2		1,732	57.8	1,253	72.3	479	27.7	
Drinking frequency							0.0006							0.0805
Frequently	1,069	13.1	701	65.6	368	34.4		402	13.4	264	65.7	138	34.3	
Occasionally	4,297	52.7	3,058	71.2	1,239	28.8		1,908	63.7	1,355	71.0	553	29.0	
None	2,781	34.1	1,989	71.5	792	28.5		685	22.9	470	68.6	215	31.4	
Physical activity							<0.0001							0.0362
5–7 days a week	1,813	22.3	1,307	72.1	506	27.9		285	9.5	211	74.0	74	26.0	
1–4 days a week	4,330	53.1	3,104	71.7	1,226	28.3		1,370	45.7	973	71.0	397	29.0	
None	2,004	24.6	1,337	66.7	667	33.3		1,340	44.7	905	67.5	435	32.5	
Stress							0.0878							0.6335
High	3,241	39.8	2,246	69.3	995	30.7		1,963	65.5	1,369	69.7	594	30.3	
Low	3,272	40.2	2,349	71.8	923	28.2		784	26.2	553	70.5	231	29.5	
None	1,634	20.1	1,153	70.6	481	29.4		248	8.3	167	67.3	81	32.7	
Depression							0.0041							0.0562
Yes	2,857	35.1	2,072	72.5	785	27.5		1,764	58.9	1,254	71.1	510	28.9	
No	5,290	64.9	3,676	69.5	1,614	30.5		1,231	41.1	835	67.8	396	32.2	
Year							0.3385							0.0022
2017	2,754	33.8	1,931	70.1	823	29.9		853	28.5	620	72.7	233	27.3	
2018	2,571	31.6	1,842	71.6	729	28.4		1,040	34.7	742	71.3	298	28.7	
2019	2,822	34.6	1,975	70.0	847	30.0		1,102	36.8	727	66.0	375	34.0	
Total	8,147	100.0	5,748	70.6	2,399	29.4		2,995	100.0	2,089	69.7	906	30.3	

* *P-values were obtained by t-test or Chi-square test*.

Table 2 presents the logistic regression analysis results adjusted for all the covariates. Adolescents having seen GHWL were more likely to attempt to quit smoking in the case of both boys and girls. Compared with those of the reference group (having not seen GHWL), the ORs (95% CIs) for attempts to quit smoking in the group with having seen GHWL were as follows: OR = 1.71 (95% CI, 1.48–2.00) in boys and OR = 1.74 (95% CI, 1.33–2.28) in girls.

**Table 2:** Odds ratio for attempts to quit smoking.

**Variables**	**Attempts to quit smoking**			
	**Boys**		**Girls**	
	**OR**	**95% CI**	**OR**	**95% CI**
Graphic health warning labels				
Have not seen	1.00		1.00	
Have seen	1.72	(1.48–2.00)	1.74	(1.33–2.28)
Smoking amount per day				
≤ 1	1.00		1.00	
2~9	1.79	(1.57–2.05)	1.65	(1.35–2.02)
≥10	1.21	(1.03–1.42)	1.07	(0.80–1.43)
Smoking onset				
Elementary school	1.11	(0.92–1.33)	1.23	(0.90–1.68)
Middle school	1.21	(1.03–1.42)	1.38	(1.08–1.75)
High school	1.00		1.00	
Passive smoking at home				
Yes	1.00		1.00	
No	1.09	(0.97–1.21)	1.13	(0.94–1.36)
Age				
12	1.00		1.00	
13	1.20	(0.67–2.12)	1.98	(0.88–4.47)
14	1.14	(0.67–1.97)	1.95	(0.90–4.26)
15	1.24	(0.72–2.14)	1.82	(0.82–4.03)
16	1.26	(0.71–2.23)	1.95	(0.83–4.57)
17	1.03	(0.58–1.82)	1.66	(0.71–3.88)
18	0.98	(0.55–1.74)	1.63	(0.68–3.88)
Region				
Metropolitan	1.00		1.00	
Urban	1.05	(0.94–1.17)	1.11	(0.92–1.34)
Rural	1.21	(0.98–1.48)	1.10	(0.77–1.57)
Grade				
Middle school	1.28	(0.99–1.67)	1.06	(0.71–1.59)
High school	1.00		1.00	
Living with family				
Yes	1.26	(1.03–1.53)	0.99	(0.72–1.38)
No	1.00		1.00	
Household income				
Low	1.08	(0.93–1.26)	1.02	(0.79–1.30)
Medium	1.01	(0.89–1.14)	0.93	(0.75–1.16)
High	1.00		1.00	
School record				
Good	1.00		1.00	
Average	1.15	(0.99–1.34)	1.21	(0.92–1.59)
Poor	1.19	(1.04–1.35)	1.35	(1.08–1.69)
Drinking frequency				
Frequently	1.00		1.00	
Occasionally	1.22	(1.03–1.43)	1.26	(0.96–1.65)
Never	1.29	(1.08–1.54)	1.22	(0.90–1.67)
Physical activity				
5–7 days a week	1.26	(1.08–1.47)	1.27	(0.92–1.75)
1–4 days a week	1.27	(1.12–1.44)	1.23	(1.02–1.49)
None	1.00		1.00	
Stress				
High	1.00		1.00	
Low	1.15	(1.01–1.30)	1.04	(0.83–1.30)
None	1.17	(1.00–1.36)	1.08	(0.77–1.51)
Depression				
Yes	1.00		1.00	
No	0.81	(0.72–0.92)	0.81	(0.67–0.99)

Table 3 presents the multiple logistic regression analysis result of the relationship between perceptions of smoking after exposure to GHWL and attempts to quit smoking controlling for all covariates, as shown in Table 2. The group that reported having not seen GHWL was set as the reference group. Students who thought about health risks of smoking after exposure to GHWL were more likely to attempt to quit smoking than students who thought it was not harmful [boys, OR = 1.86 (95% CI, 1.60–2.16) in recognition of health risks, OR = 1.15 (95% CI, 0.95–1.40) in no recognition of health risks; girls, OR = 1.85 (95% CI, 1.41–2.43) in recognition of health risks, OR = 1.07 (95% CI, 0.74–1.457) in no recognition of health risks]. Additionally, the probability of attempting to quit smoking was higher in the adolescents who thought about willingness to quit smoking after having seen GHWL than those who did not [boys, OR = 2.03 (95% CI, 1.74–2.36); girls, OR = 2.04 (95% CI, 1.55–2.68) in the willingness to quit smoking group, and boys, OR = 1.05 (95% CI, 0.87–1.25); girls, OR = 0.96 (95% CI, 0.70–1.33) in the unwillingness to quit smoking group, respectively].

**Table 3:** Odds ratio for attempts to quit smoking according to perceptions through GHWL.

**Variables**	**Attempts to quit smoking**			
	**Boys**		**Girls**	
	OR*	**95% CI**	OR^*^	**95% CI**
Recognition of health risks				
Yes (*n* = 5,985/2,397)	1.86	(1.60–2.16)	1.85	(1.41–2.43)
No (*n* = 1,053/282)	1.15	(0.95–1.40)	1.07	(0.74–1.57)
Have not seen (*n* = 1,109/316)	1.00		1.00	
Willingness to quit smoking				
Yes (*n* = 5,985/2,397)	2.03	(1.74–2.36)	2.04	(1.55–2.68)
No (*n* = 1,616/532)	1.05	(0.87–1.25)	0.96	(0.70–1.33)
Have not seen (*n* = 1,109/316)	1.00		1.00	

* *OR adjusted for all sociodemographic, economic,and health-related factors considered in the study*.

Table 4 outlines the results of the subgroup analysis regarding the effects of smoking-related factors including smoking amount per day, age of smoking onset, secondhand smoking at home; and health-related factors including drinking frequency and physical activity in an attempt to quit smoking.

**Table 4:** Results of subgroup analysis for the association between GHWL and attempts to quit smoking.

**Variables**	**Attempts to quit smoking**					
	**Boys**			**Girls**		
	**No**	**Yes**		**No**	**Yes**	
	**OR**	**OR**	**95% CI**	**OR**	**OR**	**95% CI**
Smoking amount per day						
≤ 1	1.00	1.63	(1.28–2.07)	1.00	2.33	(1.56–3.49)
2~9	1.00	1.99	(1.57–2.52)	1.00	0.95	(0.57–1.60)
≥10	1.00	1.44	(1.04–1.98)	1.00	2.78	(1.26–6.15)
Smoking onset						
Elementary school	1.00	2.05	(1.54–2.72)	1.00	2.09	(1.14–3.82)
Middle school	1.00	1.76	(1.44–2.14)	1.00	1.58	(1.08–2.30)
High school	1.00	1.28	(0.86–1.90)	1.00	1.93	(1.04–3.58)
Passive smoking at home						
Yes	1.00	1.60	(1.27–2.03)	1.00	1.63	(1.11–2.39)
No	1.00	1.79	(1.48–2.17)	1.00	1.90	(1.31–2.78)
School record						
Good	1.00	1.92	(1.45–2.54)	1.00	4.38	(2.52–7.62)
Average	1.00	1.55	(1.14–2.10)	1.00	1.11	(0.52–2.37)
Poor	1.00	1.73	(1.39–2.17)	1.00	1.40	(0.97–2.03)
Drinking frequency						
Frequently	1.00	2.34	(1.57–3.49)	1.00	2.47	(1.20–5.10)
Occasionally	1.00	1.63	(1.30–2.04)	1.00	1.29	(0.89–1.88)
Never	1.00	1.62	(1.27–2.06)	1.00	2.84	(1.64–4.90)
Physical activity						
5–7 days a week	1.00	2.13	(1.54–2.95)	1.00	2.01	(0.85–4.74)
1–4 days a week	1.00	1.63	(1.33–2.01)	1.00	2.29	(1.54–3.40)
None	1.00	1.67	(1.25–2.22)	1.00	1.14	(0.74–1.77)
Stress						
High	1.00	1.76	(1.39–2.23)	1.00	1.65	(1.18–2.32)
Low	1.00	1.69	(1.32–2.16)	1.00	1.27	(0.70–2.30)
None	1.00	1.73	(1.24–2.40)	1.00	3.55	(1.41–8.91)
Depression						
Yes	1.00	1.69	(1.30–2.19)	1.00	2.10	(1.46–3.03)
No	1.00	1.78	(1.49–2.13)	1.00	1.41	(0.94–2.11)

In participants who had seen GHWL, boys and girls who smoked 2–9 cigarettes per day [OR = 1.99 (95% CI = 1.57–2.52)] and more than 10 cigarettes per day [OR = 2.78 (95% CI = 1.26–6.15)], respectively, were most likely to attempt to quit smoking. Both boys and girls who started smoking at elementary school were more likely to try to quit smoking compared to those who started smoking later [OR = 2.05 (95% CI = 1.54–2.72) in boys; OR = 2.09 (95% CI = 1.14–3.82) in girls]. Boys had higher likelihood of trying to quit smoking when they drank alcohol frequently [OR = 2.34 (95% CI = 1.57–3.49)], whereas girls had higher odds of trying to quit smoking when they did not drink alcohol at all [OR = 2.84 (95% CI = 1.64–4.90)]. Participants who performed physical activity for more than 60 min a day had higher odds of attempting to stop smoking than those who did not perform any physical activity [boys, OR = 2.13 (95% CI = 1.54–2.95) in 5–7 days a week; girls, OR = 2.29 (95% CI = 1.54–3.40) in 1–4 days per week].

## Discussion

GHWL is a worldwide policy for smoking cessation to prevent adolescents' smoking initiation and is potentially effective in influencing smoking attitudes and behaviors.

This study was conducted to identify the association between GHWL and attempts to quit smoking in Korean adolescents using representative data recorded via the KYRBWS. We also conducted a subgroup analysis based on amount smoked per day, age of smoking onset, secondhand smoking at home, drinking frequency, and physical activity; which are factors related to smoking habits.

We observed that adolescent smokers who reported exposure to GHWL were more likely to attempt to quit smoking compared to those who did not. Boys and girls who saw GHWL were 1.72 times and 1.74 times more likely to try to quit smoking, respectively. Additionally, in both sexes, there was an increased attempt to stop smoking in those who thought about its harm and the willingness to quit after exposure to GHWL. The findings of this study were consistent with those of earlier studies, which suggests that GHWL are positively associated with reducing smoking in adolescents (16, 21–23). Other studies conducted on Korean adolescents or adults also revealed that GHWL can be an effective way to prevent smoking (16, 24–27). Smokers who noticed the warnings were significantly more likely to disclose associated health risks.

Another study showed that GHWL were ineffective in curbing smoking rates and preventing smoking and smoking initiation in adolescents (28). To reduce the smoking rate of regular adolescent smokers, it is necessary to evaluate and implement effective tobacco control policies, particularly cigarette prices; habitual smokers are more likely to remain persistent smokers compared with occasional smokers among Korean adolescents (29).

A prior study also reported that adolescents who were occasionally or regularly exposed to secondhand smoking at home were less likely to attempt to cease smoking (30). Among adolescents (in grades 7–12), the prevalence of secondhand smoke exposure at home was higher among female students (32.0%) than male students (29.5%) (31). Incidentally, another study reported decreased use of cigarettes among adolescents who had current smokers among family members (32). Moreover, smoking was more prevalent in adolescents who drank alcohol, specifically among those treated for alcohol use disorders (33). Adolescents were more likely to purchase cigarettes when they had problematic drinking within 1 year (32). Several studies have reported that the experience of binge drinking and its frequency was associated with buying cigarettes (34, 35). A previous study also indicated that adolescents—for whom the daily amount of cigarette smoking was higher—were more likely to succeed in their attempts to obtain cigarettes, when they had current smokers among close friends (32). Since family, friends, and surrounding environments easily influence adolescence's behavior, adolescents could be likely to start smoking by mimicking acquaintances who smoke (36).

To protect youth from tobacco products, Korea has implemented several laws and regulations such as prohibiting the sale of tobacco products to minors, requiring age verification when selling tobacco products, and designating school facilities as smoking-free areas (32). Nevertheless, cigarette purchases are easy enough in Korea that more than 70% of adolescents can buy cigarettes when they attempt to obtain them (32). Also, Korean government increased cigarette prices from 2,500 won (US$2.09) to 4,500 won (US$3.76) in 2015, but they are still cheaper than other countries (37, 38). This suggests that comprehensive control of tobacco including non-price strategies such as advertisement restrictions and health warnings should be further reinforced to regulate adolescents access to tobacco.

This study has several limitations that should be considered when interpreting the results. First, we used cross-sectional data; thus, a clear causal relationship between GHWL and attempts to quit smoking could not be inferred. Second, since smoking-related variables were evaluated based on the participants' memory, there is a likelihood of recall bias. Third, the results were derived from self-reported data; thus, the data from participants may be subjected to response bias. As shown by a previous study in Korea, females tend to under-report their smoking status (20). Fourth, our study lacked information about individuals who successfully quit smoking. Thus, we could not assess the relationship between GHWL and smoking cessation success. Additionally, owing to the limited information on the number of attempts to quit smoking and the success, further investigations related to the success of smoking cessation are needed.

Despite these limitations, our study has some strengths. We used nationwide representative data that were suitable for conducting Korean studies. Since the KYRBWS is representative of the overall adolescent student population in grades 7–12, the results in our study can be generalized to the overall Korean adolescent population. Furthermore, since the KYRBWS was a web-based anonymous survey, obtaining honest responses was relatively likely. The results from our study can be used as a baseline for developing effective policies to prevent adolescent smoking. Clearly, the present study is meaningful because its results support and add to the findings of previous studies by demonstrating that GHWL can help in preventing smoking in adolescents. Society, particularly schools, should continue to educate adolescent smokers through improved smoking cessation programs with constant monitoring. Nevertheless, we recommend that government and public health professionals recognize the need to empower adolescents to quit smoking through intensive efforts to reduce adolescents' tobacco use by developing and promoting GHWL and other tobacco control policies such as media campaigns, school-based education, legislation to ban smoking, and public service announcements.

## Conclusion

This study demonstrated a significant association between GHWL and attempts to quit smoking in Korean adolescents. The correlation was greater in the case of adolescents who thought about the risks associated with smoking and willingness to quit, after their exposure to GHWL. These findings indicate that GHWL could play a crucial role to effectively prevent youth from smoking initiation and facilitate smoking cessation. Notably, our research can help health policy makers and experts in implementing smoking prevention and cessation strategies for managing adolescents' health by increasingly utilizing GHWL as an effective means to prevent adolescents from smoking.

## Data Availability Statement

The datasets presented in this study can be found in online repositories. The names of the repository/repositories and accession number(s) can be found below: the data of Korea Youth Risk Behavior Web-based Survey (KYRBWS) are publicly available through the Korea Centers for Disease Prevention and Control website (https://www.kdca.go.kr/yhs/).

## Ethics Statement

Ethical review and approval was not required for the study on human participants in accordance with the local legislation and institutional requirements. Written informed consent to participate in this study was provided by the participants' legal guardian/next of kin.

## Author Contributions

HJ designed the study, collected the data, performed the statistical analysis, and drafted the manuscript. HJ, JJ, SK, E-CP, and S-IJ contributed to the discussion, reviewed, and edited the manuscript. S-IJ is the guarantor of this work and as such, has full access to all the study data and assumes the responsibility for the integrity of the data, and accuracy of the data analysis. All authors have approved the final article.

## Funding

This research was supported by a grant of the Korea Health Technology R&D Project through the Korea Health Industry Development Institute (KHIDI), funded by the Ministry of Health and Welfare, Republic of Korea (Grant Number: HI20C1130).

## Conflict of Interest

The authors declare that the research was conducted in the absence of any commercial or financial relationships that could be construed as a potential conflict of interest.

## Publisher's Note

All claims expressed in this article are solely those of the authors and do not necessarily represent those of their affiliated organizations, or those of the publisher, the editors and the reviewers. Any product that may be evaluated in this article, or claim that may be made by its manufacturer, is not guaranteed or endorsed by the publisher.

## References

[1] Oh SS Jang J-E Lee D-W Park E-C Jang S-I. Cigarette type or smoking history: which has a greater impact on the metabolic syndrome and its components? Sci Rep. (2020) 10:1–8. 10.1038/s41598-020-67524-2 32591636 PMC7319978

[2] World Health Organization. WHO Report on the Global Tobacco Epidemic 2019: Offer Help to Quit Tobacco Use. World Health Organization (2019).

[3] Lee HJ Jang J Choi D-W Chae W Park E-C Jang S-I. Association between change in lifestyle and cognitive functions among elderly Koreans: findings from the Korean longitudinal study of aging (2006–2016). BMC Geriatr. (2020) 20:1–12. 10.1186/s12877-020-01693-7 32867702 PMC7457530

[4] Jang BN Lee HJ Joo JH Park E-C Jang S-I. Association between health behaviours and depression: findings from a national cross-sectional study in South Korea. BMC Psychiatry. (2020) 20:1–9. 10.1186/s12888-020-02628-7 32408865 PMC7227033

[5] World Health Organization. Tobacco. Available online at: http://www.who.int/mediacentre/factsheets/fs339/en/ (accessed September 25, 2021).

[6] Kim E-M Park E Kim H. Sex differences in multilevel factors of smoking experimentation and age of initiation in Korean adolescents. J Sch Nurs. (2020) 36:348–59. 10.1177/1059840519840805 30966858

[7] Kim HW Kang SN Lim JS Lee JA Cho H-J. Changes of cigarette smoking initiation age among south korean adults: 2007-2012. J Korean Soc Res Nicotine Tob. (2017) 8:20–8. 10.25055/JKSRNT.2017.8.1.20

[8] Riggs NR Chou C-P Li C Pentz MA. Adolescent to emerging adulthood smoking trajectories: when do smoking trajectories diverge, and do they predict early adulthood nicotine dependence? Nicotine Tob Res. (2007) 9:1147–54. 10.1080/14622200701648359 17978988

[9] World Health Organization. WHO Report on the Global Tobacco Epidemic 2015: Raising Taxes on Tobacco. World Health Organization (2015). ISBN: 9241509120.

[10] Crawford MA Balch GI Mermelstein R. Responses to tobacco control policies among youth. Tob Control. (2002) 11:14–9. 10.1136/tc.11.1.14 11891362 PMC1747664

[11] Hawkins SS Bach N Baum CF. Impact of tobacco control policies on adolescent smoking. J Adolesc Health. (2016) 58:679–85. 10.1016/j.jadohealth.2016.02.014 27151762 PMC4877221

[12] Hammond D Fong GT McNeill A Borland R Cummings KM. Effectiveness of cigarette warning labels in informing smokers about the risks of smoking: findings from the International Tobacco Control (ITC) four country survey. Tob Control. (2006) 15(Suppl. 3):iii19–25. 10.1136/tc.2005.012294 16754942 PMC2593056

[13] Strahan EJ White K Fong GT Fabrigar LR Zanna MP Cameron R. Enhancing the effectiveness of tobacco package warning labels: a social psychological perspective. Tob Control. (2002) 11:183–90. 10.1136/tc.11.3.183 12198266 PMC1759023

[14] Borland R Hill D. The path to Australia's tobacco health warnings. Addiction. (1997) 92:1151–8. 10.1111/j.1360-0443.1997.tb03674.x 9374013

[15] Fong GT Hammond D Hitchman SC. The impact of pictures on the effectiveness of tobacco warnings. Bull World Health Organ. (2009) 87:640–3. 10.2471/BLT.09.069575 19705020 PMC2733253

[16] Kang E. Assessing health impacts of pictorial health warning labels on cigarette packs in korea using DYNAMO-HIA. J Prev Med Public Health. (2017) 50:251–61. 10.3961/jpmph.17.032 28768403 PMC5541276

[17] Kim Y Choi S Chun C Park S Khang Y-H Oh K. Data resource profile: the Korea youth risk behavior web-based survey (KYRBS). Int J Epidemiol. (2016) 45:1076–1076e. 10.1093/ije/dyw070 27380796

[18] Bae J Joung H Kim JY Kwon KN Kim Y Park SW. Validity of self-reported height, weight, and body mass index of the Korea youth risk behavior web-based survey questionnaire. J Prev Med Public Health. (2010) 43:396–402. 10.3961/jpmph.2010.43.5.396 20959710

[19] Bae J Joung H Kim JY Kwon KN Kim YT Park SW. Test-retest reliability of a questionnaire for the Korea youth risk behavior web-based survey. J Prev Med Public Health. (2010) 43:403–10. 10.3961/jpmph.2010.43.5.403 20959711

[20] Kang HG Kwon KH Lee IW Jung B Park E-C Jang S-I. Biochemically-verified smoking rate trends and factors associated with inaccurate self-reporting of smoking habits in Korean women. Asian Pac J Cancer Prev. (2013) 14:6807–12. 10.7314/APJCP.2013.14.11.6807 24377610

[21] White V Bariola E Faulkner A Coomber K Wakefield M. Graphic health warnings on cigarette packs: how long before the effects on adolescents wear out? Nicotine Tob Res. (2014) 17:776–83. 10.1093/ntr/ntu184 25239958

[22] Hammond D. Health warning messages on tobacco products: a review. Tob Control. (2011) 20:327–37. 10.1136/tc.2010.037630 21606180

[23] Hiilamo H Crosbie E Glantz SA. The evolution of health warning labels on cigarette packs: the role of precedents, and tobacco industry strategies to block diffusion. Tob Control. (2014) 23:e2–e2. 10.1136/tobaccocontrol-2012-050541 23092884 PMC3725195

[24] Hwang J-e Cho S-i. The association between new graphic health warning labels on tobacco products and attitudes toward smoking among south Korean adolescents: a national cross-sectional study. BMC Public Health. (2020) 20:1–10. 10.1186/s12889-020-08638-0 32448193 PMC7245864

[25] Seo HG Cheong YS Myung SK Kim Y Lee WB Fong GT. Smoking-related characteristics in Korean adult smokers: findings from the 2005 international tobacco control policy evaluation survey-korea. J Korean Acad Fam Med. (2008) 29:844–53.

[26] Lee SM Chun S Lee JS. The role of negative emotions pre- and post-implementation of graphic health warnings: longitudinal evidence from South Korea. Int J Environ Res Public Health. (2020) 17:5393. 10.3390/ijerph17155393 32727019 PMC7432201

[27] Jung S Seo S-I Oh S-W Cho S-I Hwang S-S. Systematic review of graphic warning on cigarette packages for reducing tobacco use in Korea. J Korean Soc Res Nicotine Tob. (2018) 9:4–17. 10.25055/JKSRNT.2018.9.1.4

[28] Cho KS Shin YJ A A survey on the effect of cigarette warning labels. J Korean Acad Fam Med. (2006) 27:128–35.

[29] Lee YS Kim H-S Kim H-D Yoo K-B Jang S-I Park E-C. Is a Price increase policy enough for adolescent smokers?: Factors affecting the effectiveness of increasing cigarette prices among Korean adolescent smokers. Nicotine Tob Res. (2016) 18:2013–9. 10.1093/ntr/ntw122 27117284

[30] Jeong W Kim YK Joo JH Jang S-I Park E-C. The association of smoking exposure at home with attempts to quit smoking and cessation success: a survey of South Korean adolescents who smoke. Int J Environ Res Public Health. (2020) 17:4129. 10.3390/ijerph17114129 32531888 PMC7312504

[31] Choi S Kim Y Park S Lee J Oh K. Trends in cigarette smoking among adolescents and adults in South Korea. Epidemiol Health. (2014) 36:e2014023. 10.4178/epih/e2014023 25358464 PMC4371385

[32] Kang SY Lee JA Cho H-J. Trends in the ease of cigarette purchase among Korean adolescents: evidence from the Korea youth risk behavior web-based survey 2005–2016. BMC Public Health. (2018) 18:1242. 10.1186/s12889-018-6151-9 30404618 PMC6222989

[33] Myers MG Kelly JF. Cigarette smoking among adolescents with alcohol and other drug use problems. Alcohol Res Health. (2006) 29:221–7. 17373413 PMC1931414

[34] Vu M Leatherdale ST Ahmed R. Examining correlates of different cigarette access behaviours among Canadian youth: data from the Canadian youth smoking survey (2006). Addict Behav. (2011) 36:1313–6. 10.1016/j.addbeh.2011.07.010 21821363

[35] Hughes SK Hughes K Atkinson AM Bellis MA Smallthwaite L. Smoking behaviours, access to cigarettes and relationships with alcohol in 15-and 16-year-old schoolchildren. Eur J Public Health. (2011) 21:8–14. 10.1093/eurpub/ckp234 20145050

[36] Conrad KM Flay BR Hill D. Why children start smoking cigarettes: predictors of onset. Br J Addict. (1992) 87:1711–24. 10.1111/j.1360-0443.1992.tb02684.x 1490085

[37] Lee B Seo D-C. Effects of an 80% cigarette price increase on quit attempts, successful quitting and smoking intensity among Korean adult smokers: results from nationally representative longitudinal panel data. Tob Control. (2021) 30:336. 10.1136/tobaccocontrol-2019-055518 32269171

[38] Cherukupalli R. Korea's 2015 cigarette tax increases. Tob Control. (2016) 25:123–4. 10.1136/tobaccocontrol-2014-052104 25673328

